# Whole-Genome Sequence of the Potentially Hypertransmissible Multidrug-Resistant *Mycobacterium tuberculosis* Beijing Strain OM-V02_005

**DOI:** 10.1128/genomeA.00608-13

**Published:** 2013-08-08

**Authors:** Yoshitaka Tateishi, Aki Tamaru, Yoshitoshi Ogura, Mamiko Niki, Takayuki Wada, Taro Yamamoto, Kazuto Hirata, Tetsuya Hayashi, Sohkichi Matsumoto

**Affiliations:** Department of Bacteriology, Graduate School of Medicine, Osaka City University, Osaka, Japana; National Hospital Organization, Toneyama Hospital, Osaka, Japanb; Osaka Prefectural Institute of Public Health, Osaka, Japanc; Department of Life Science, Frontier Science Research Center, University of Miyazaki, Miyazaki, Japand; Department of International Health, Institute of Tropical Medicine, Nagasaki University, Nagasaki, Japane; Department of Respiratory Medicine, Graduate School of Medicine, Osaka City University, Osaka, Japanf

## Abstract

We report the draft genome sequence of *Mycobacterium tuberculosis* Beijing strain OM-V02_005, which exhibits possible hypertransmissible characteristics among the population of patients with multidrug-resistant tuberculosis in Osaka Prefecture, the largest urban area in western Japan.

## GENOME ANNOUNCEMENT

The *Mycobacterium tuberculosis* Beijing strain is a causative agent of tuberculosis with increasing prevalence, and it is assumed to have a considerable impact on resistance to major antituberculosis drugs. We identified the most prevalent cluster (OM-V02) among the population of multidrug-resistant tuberculosis (MDR-TB) patients in Osaka Prefecture, the largest urban area in western Japan (1). To elucidate the underlying genetic characteristics of the transmissibility and drug resistance of this cluster, specifically found in MDR-TB patients, we performed whole-genome sequencing of one representative strain, OM-V02_005.

We sequenced OM-V02_005 genomic DNA on a 454 GS-FLX Titanium Sequencer (Roche) and assembled the reads using Newbler version 2.3. A total of 510,688 reads was generated, with an average read length of 439 bp, yielding a total sequence of 224,340,568 bp.

The assembled sequences contained 134 contigs, and the length of all contigs combined was 4,321,797 bp, with a G+C ratio of 65.5%. The average coverage depth was 52×, the *N*_50_ contig size was 68,368 bp, the average contig was 32,252 bp long, and the longest contig was 184,273 bp.

Genome annotation was performed using GeneMark.hmm for prokaryotes (http://exon.gatech.edu/gmhmm2_prok.cgi), xBASE (http://www.xbase.ac.uk/annotation/) for coding sequence genes, and tRNAscan-SE 1.21 (http://lowelab.ucsc.edu/tRNAscan-SE/) and RNAmmer 1.2 (http://www.cbs.dtu.dk/services/RNAmmer/) for RNA genes. OM-V02_005 has 3,888 coding-sequence genes, 3 rRNAs (in a single operon), and 52 tRNA genes.

We compared the OM-V02_005 sequence with that of other representative *M. tuberculosis* strains: H37Rv (GenBank accession no. AL123456.2), CDC1551 (AE000516), F11 (CP000717), CCDC5079 (CP001641), and CCDC5080 (CP001642). *M. tuberculosis* CCDC5079 is a drug-sensitive strain and CCDC5080 is a multidrug-resistant Beijing strain sequenced in China (2). The reciprocal best-hit BLAST approach revealed that OM-V02_005 shares 90.0%, 92.7%, 90.0%, 92.7%, and 88.7% of its coding sequences with *M. tuberculosis* H37Rv, CDC1551, F11, CCDC5079, and CCDC5080, respectively.

OM-V02_005 has 89 specific genes. Most of these were classified as genes for hypothetical proteins, but genes for both a dehydrogenase enzyme and the Tuf-like elongation factor Tu showed specific sequence alignment between OM-V02_005 and a clinical strain of *M. tuberculosis* T46 isolated in San Francisco from a Filipino patient.

We investigated single nucleotide polymorphisms (SNPs) using SNPs Finder (http://snpsfinder.lanl.gov/). OM-V02_005 has 270 specific SNPs, of which 169 were nonsynonymous mutations, 66 were synonymous mutations, and 26 were mutations in promoter regions. In addition to our previous report of the mutations in *rrs*, *rpoB*, *katG*, *gyrA*, *embB*, and *pncA* (1), mutations were found in *rrl* as I236T and in *gidB* as A413E, respectively. No significant mutations were found in *inhA*, *oxyS*, *iniB*, *iniA*, *iniC*, *furA*, and *rpsL* (Rv0682). Nonsynonymous mutations include regulatory proteins for growth/dormancy regulation (*pknA*, *mprA*, and *rpfA*), intermediary metabolism and regulation (*coaA*, *ribH*, *tal*, and *bfrB*), virulence genes (*clpB and mce3F*), lipid metabolism (*ppsB* and *ppsD*), and cell wall proteins (*lprK/mceIE*, *mmpL1*, *mmpL2*, and *dppA*). The OM-V02_005 sequence data will provide an invaluable resource to elucidate the underlying mechanisms of transmissibility and drug resistance acquisition of *M. tuberculosis*.

### Nucleotide sequence accession numbers.

The whole genome sequence of OM-V02_005 has been deposited in DDBJ/EMBL/GenBank under the accession numbers BARZ01000001 through BARZ01000121.
